# Thrombocytopenia, anasarca, and renal insufficiency as severe and rare complications of Hodgkin lymphoma: a case report

**DOI:** 10.1186/s13256-023-03776-6

**Published:** 2023-02-21

**Authors:** Tatsuya Kikuchi, Yoshinori Tanaka, Kouichi Ichimura, Hiroyuki Okada, Ryoichi Okamoto

**Affiliations:** 1 Department of Internal Medicine, Hiroshima City Hiroshima Citizens Hospital, Hiroshima, Japan; 2 Department of Gastroenterology, Okayama University Hospital, Okayama, Japan; 3 Department of Hematology, Hiroshima City Hiroshima Citizens Hospital, Hiroshima, Japan; 4 Department of Pathology, Hiroshima City Hiroshima Citizens Hospital, Hiroshima, Japan

**Keywords:** Hodgkin lymphoma, Hemophagocytic syndrome, TAFRO syndrome

## Abstract

**Background:**

Patients with Hodgkin lymphoma exhibit various clinical presentations. Needle biopsy of the lymph nodes is a minimally invasive procedure and a useful diagnostic method for malignant lymphomas. However, at times it is difficult to differentiate malignant lymphomas from reactive lymph node changes using a small amount of biopsy material.

**Case presentation:**

A 77-year-old Japanese man was referred to the emergency department of our hospital owing to high fever and disturbance of consciousness. We diagnosed sepsis due to an acute biliary tract infection because he presented with Charcot’s triad—fever, jaundice, and right-sided abdominal pain. However, he did not respond well to antimicrobial therapy and his high fever persisted. Considering the swelling of the right cervical, mediastinal, and intraperitoneal lymph nodes and splenomegaly detected on computed tomography, a differential diagnosis of malignant lymphoma was needed. Hence, we performed a needle biopsy of the right cervical lymph node; however, the amount of sample obtained was insufficient in establishing a definitive diagnosis of malignant lymphoma. Furthermore, during hospitalization, the patient developed thrombocytopenia, anasarca, and renal insufficiency. These symptoms seemed to be the typical signs of the thrombocytopenia, anasarca, fever, reticulin fibrosis or renal insufficiency, and organomegaly syndrome. Next, an external incisional mass biopsy of the right cervical lymph node was performed, which helped identify Hodgkin and Reed–Sternberg cells. Collectively, we established a definitive diagnosis of Hodgkin lymphoma with lymphoma-associated hemophagocytic syndrome.

**Conclusions:**

This case highlights the importance of performing an external incisional mass biopsy of the lymph nodes for the early diagnosis and treatment, if malignant lymphoma is strongly suspected.

## Background

Patients with Hodgkin lymphoma present with various clinical symptoms. In some patients with Hodgkin lymphoma, the initial symptom is painless and elastic cervical lymphadenopathy, while in others it may include axillary lymphadenopathy, inguinal lymphadenopathy, or splenomegaly. In addition, patients sometimes present with B symptoms including fever, night sweats, and weight loss. In general, leukemia is rarely observed, and the prognosis is relatively good if patients receive appropriate treatment [1, 2]. It is well documented that lymphoma-associated hemophagocytic syndrome (LAHS) is a common complication of non-Hodgkin lymphoma. However, it is extremely rare in Hodgkin lymphoma.

Patients with sepsis often experience severe complications, such as thrombocytopenia, anasarca, and renal insufficiency [3, 4]. These symptoms are similar to the typical symptoms of the thrombocytopenia, anasarca, fever, reticulin fibrosis or renal insufficiency, and organomegaly (TAFRO) syndrome, as originally reported by Takai *et al.* in 2010 [5]. TAFRO syndrome is categorized as a subtype of idiopathic multicentric Castleman disease (iMCD); however, its etiology remains unclear and controversial. Compared with that of other iMCD subtypes, the clinical course of TAFRO syndrome is particularly progressive. Diagnostic criteria and disease severity classifications have been gradually established [6–10]. Malignant lymphomas should be excluded from the diagnosis of TAFRO syndrome.

## Case presentation

A 77-year-old Japanese man was on medication for hypertension and dyslipidemia. His family and psychosocial history were unremarkable. Over a year before presentation, he had noticed a mass on the right side of his neck. He visited his doctor every 3 months for follow-up. However, no pathological examination was performed, because the maximum diameter of the right cervical lymph node was approximately 20 mm and it remained almost unchanged. A month before presentation, the patient experienced loss of appetite, and a week before presentation, he had persistent fever with a body temperature above 38 °C. He underwent polymerase chain reaction testing for coronavirus disease 2019 (COVID-19), and the result was negative. Subsequently, his general condition gradually deteriorated and he was transferred to the emergency department of our hospital because of high fever and disturbance of consciousness.

**Fig. 1:**
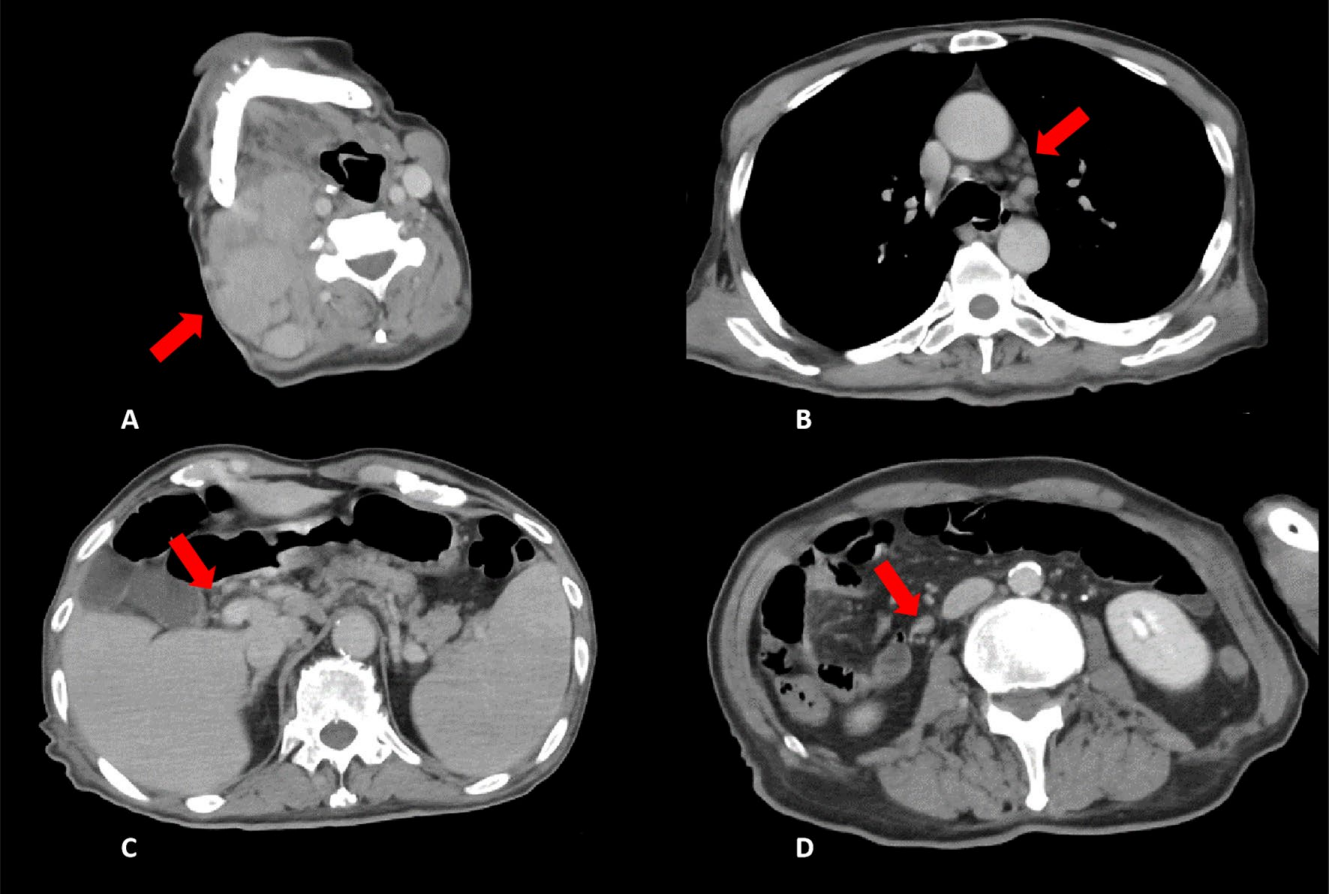
Contrast-enhanced computed tomography (CT) images demonstrated swelling of multiple lymph nodes. Red arrows indicate **A** right cervical lymph nodes, **B** mediastinal lymph nodes, and **D** intraperitoneal lymph nodes. **C** Mild dilatation of the extrahepatic bile duct with contrast enhancement and splenomegaly

His vital signs at the time of visit were as follows: Glasgow Coma Scale score, E4V5M6; body temperature, 39.9 °C; blood pressure, 122/74 mmHg; heart rate, 148 beats per minute, regular; respiratory rate, 24 breaths per minute; SpO2, 98% on room air respiration. We speculated that tachycardia was due to the high fever and volume depletion due to poor oral intake. Swelling of the right cervical lymph node was painless and elastic. The maximum diameter of the right cervical lymph nodes was approximately 40 mm. A heart examination revealed normal findings and the absence of a murmur, and a chest assessment revealed normal bilateral sounds. Further, an abdominal inspection revealed tenderness in the right upper abdomen. He had no edema of the extremities. Blood test results showed high total bilirubin and hepatobiliary system enzyme levels, and the following parameters were noted: peripheral white blood cell count, 8600/µL with 90.4% neutrophils and 2.9% lymphocytes; hemoglobin, 16.7 g/dL; platelet, 18.6 × 10^4^/µL; total bilirubin, 2.0 mg/dL; aspartate transaminase, 43 U/L; alanine aminotransferase, 114 U/L; alkaline phosphatase, 1055 U/L; gamma-glutamyl transpeptidase 216 U/L; lactic acid dehydrogenase, 239 U/L; C-reactive protein, 18.6 mg/dL. Contrast-enhanced computed tomography (CT) revealed mild dilatation of the extrahepatic bile duct with enhancement of the contrast effect. Considering these findings, we suspected acute inflammation of the bile duct (Fig. 1C).

We diagnosed sepsis due to an acute biliary tract infection. Magnetic resonance cholangiopancreatography was also performed, but a common bile duct stone was not observed. Transient cholestasis or biliary sludge was considered a cause of acute inflammation of the bile duct. Therefore, the patient was hospitalized immediately, and treatment with an antimicrobial agent was initiated. Cefmetazole displays a wide spectrum of activity against bacteria, including Gram-positive and Gram-negative aerobes and anaerobes, and is thus used in the treatment of intraabdominal infections. Therefore, we decided to use this antimicrobial therapy. However, the patient did not respond well, and his high fever persisted. On day 6 of hospitalization, edema at the extremities, pleural effusion, and ascites began to appear. Considering the swelling of the right cervical, mediastinal, and intraperitoneal lymph nodes (Fig. 1A, B, D) and the splenomegaly detected on CT (Fig. 1C), we considered the diagnosis of malignant lymphoma. Additional blood test results revealed an elevated soluble interleukin-2 receptor level (10,596 U/mL). We performed a needle biopsy of the right cervical lymph node, but there was no evidence of malignant lymphoma. Subsequently, we discussed the need for an external incisional mass biopsy; however, it was difficult to perform surgical intervention because of the patient’s unstable condition, including circulatory insufficiency and impaired consciousness.

**Fig. 2:**
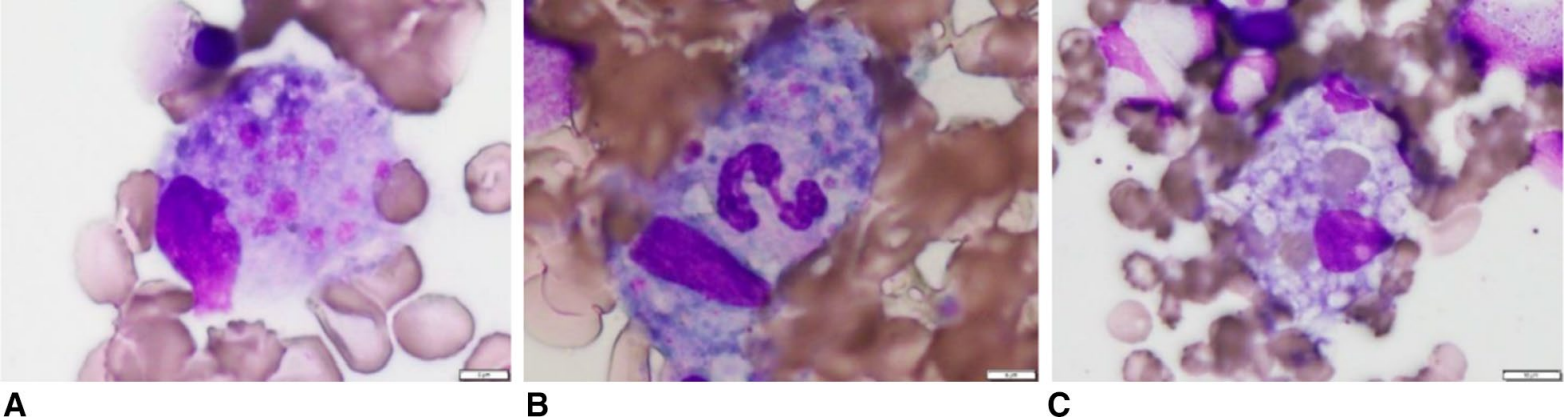
Bone marrow biopsy findings. Macrophages phagocytosed the **A** platelets, **B** neutrophils, and **C** red blood cells

**Fig. 3:**
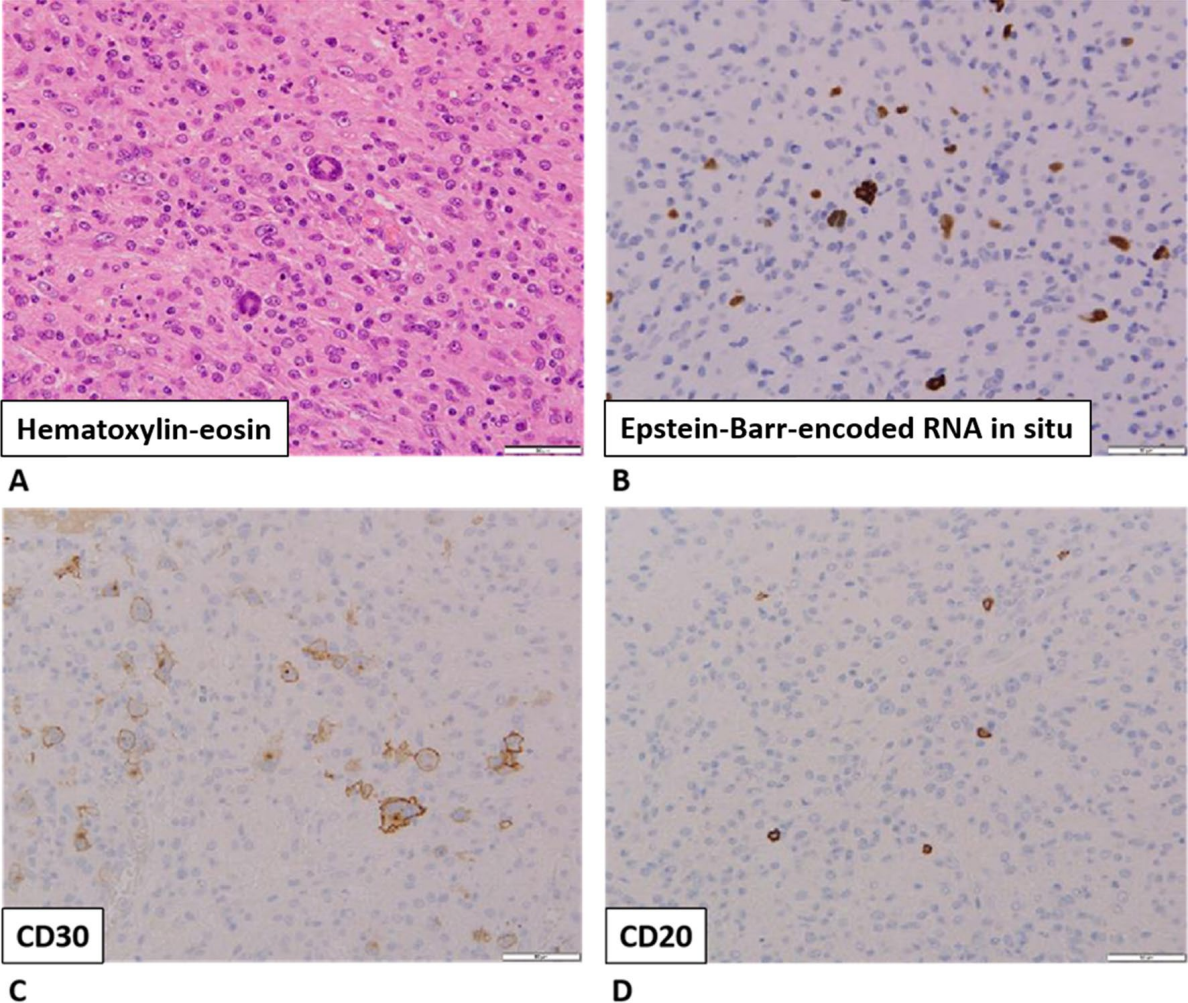
Immunohistochemical findings of an external incisional mass biopsy of the right cervical lymph node.** A** Hematoxylin-eosin staining.** B** Epstein-Barr-encoded RNA* in situ* hybridization.** C** CD30 staining.** D** CD20 staining

On day 10 of hospitalization, the patient’s general condition temporarily improved. However, thrombocytopenia, anasarca, and renal insufficiency became exacerbated. His serum creatinine level increased to 3.01 mg/dL. We initially thought that these symptoms were complications of sepsis, but they also seemed to be typical of the five signs of TAFRO syndrome. To detect reticulin fibrosis, we performed a bone marrow biopsy to confirm the diagnosis of TAFRO syndrome. Although reticulin fibrosis was not detected, increased levels of macrophages were observed (Fig. 2). An external incisional mass biopsy of the right cervical lymph node was then performed. Surprisingly, we identified Hodgkin and Reed–Sternberg cells. Immunohistochemical analysis revealed the cells to be positive for CD30 and negative for CD20, CD5, and CD10 expression. CD30 is known to be strongly expressed in mononuclear Hodgkin cells and polynuclear Reed–Sternberg cells in Hodgkin lymphoma and anaplastic large-cell lymphoma tumors, while CD20 is widely expressed as a B-cell surface antigen. It has been documented that CD5 expression is positive in chronic lymphocytic leukemia, T-cell lymphoma, and mantle cell lymphoma and that CD10 expression is positive in follicular lymphoma and Burkitt lymphoma. In addition, Epstein–Barr-encoded RNA *in situ* hybridization results were positive, as observed in Hodgkin lymphoma (Fig. 3) [11].

We established a definitive diagnosis of Hodgkin lymphoma with lymphoma-associated hemophagocytic syndrome (LAHS). Unfortunately, as the patient had multiple organ failure at the time of diagnosis, it was difficult to administer induction chemotherapy. Therefore, steroid pulse therapy was administered. However, the patient did not respond well and died on day 22 of hospitalization.

## Discussion

Hodgkin lymphoma is generally considered a slow-progressing disease that predominantly affects individuals in their 20s and 50s. LAHS is a common complication of non-Hodgkin lymphoma, such as natural killer/T-cell lymphoma and intravascular large B-cell lymphoma, but is rarely observed in Hodgkin lymphoma [12, 13]. Therefore, this case seems to be atypical for Hodgkin lymphoma because of the patient’s age and acute course of disease progression that led to multiple organ failure. To elucidate the causes of thrombocytopenia, acute liver failure, and acute renal failure, we examined the pathological anatomy of the patient.

**Fig. 4:**
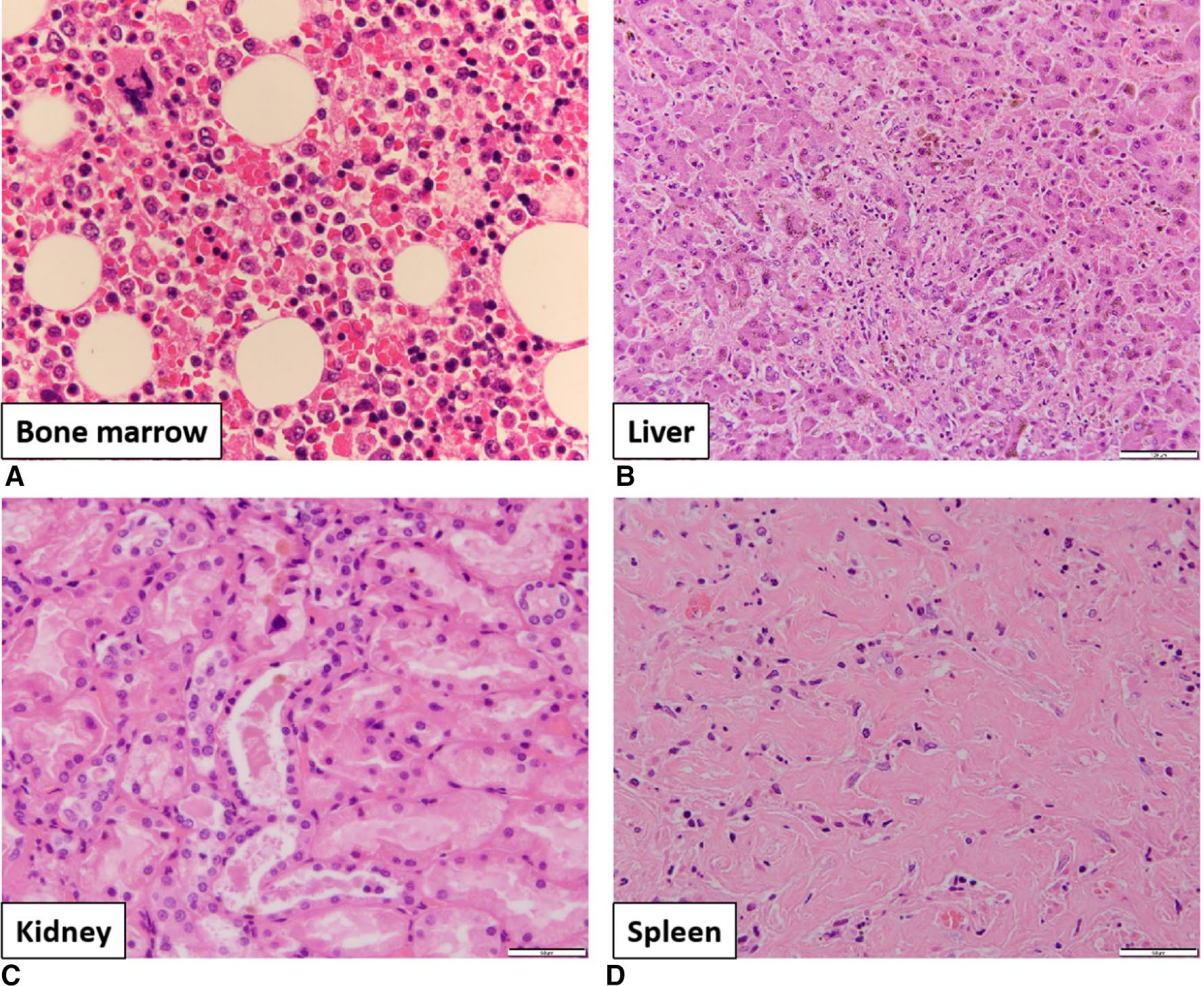
Pathological anatomy findings. **A** Hemophagocytosis in the bone marrow. **B** Cholestasis and jaundice of the intralobular bile ducts in the liver. **C** Bile casts in the renal tubule. **D** Fibrosis in the spleen

The final diagnosis was classical Hodgkin lymphoma with infiltration of the bone marrow, liver, and spleen. The main cause of thrombocytopenia was infiltration of tumor cells into the bone marrow leading to suppression of hematopoiesis and an increase in hemophagocytosis (Fig. 4A). Acute liver failure was considered to be induced by infiltration of tumor cells into the liver and cholestasis in the intralobular bile ducts (Fig. 4B). Furthermore, bile casts in the renal tubules were considered one of the causes of acute renal failure (Fig. 4C). In contrast, we observed focal necrosis, patchy fibrosis, and infiltration of tumor cells in the spleen (Fig. 4D). These findings suggest that these pathological changes occur over a long period of time. In conclusion, this was a case of classical Hodgkin lymphoma in the terminal phase.

The clinical presentation of the patient at the time of the patient’s visit was similar to that of sepsis due to an acute biliary tract infection. During hospitalization, the patient developed thrombocytopenia, anasarca, and renal insufficiency. These symptoms seemed to be the result of a cytokine storm induced by sepsis but were difficult to distinguish from other causes, such as TAFRO syndrome. There have been several case reports of patients with TAFRO syndrome successfully treated with glucocorticoid treatment or in combination with other immunosuppressants, such as rituximab and tocilizumab [14–18]. The usefulness of tocilizumab is based on the observation of elevated serum interleukin (IL)-6 levels in patients with TAFRO syndrome. However, in this case, the serum IL-6 level was within the normal range. In addition, we detected multiple adenopathies on CT, considered the diagnosis of malignant lymphoma, and performed a needle biopsy of the right cervical lymph node. However, this was insufficient to establish a definitive diagnosis for malignant lymphoma. These clinical findings make it difficult to obtain a diagnosis and initiate appropriate treatment.

Needle biopsy is a minimally invasive procedure and a useful diagnostic method for malignant lymphomas, even if it is difficult for patients to undergo surgical intervention. However, it is sometimes difficult to differentiate malignant lymphomas from reactive lymph node changes using a small amount of biopsy material. In addition, immunohistochemical analysis, as recommended in the World Health Organization classification, is important in the treatment of malignant lymphomas; however, it can be challenging if surgical specimens are not available. Roemer *et al.* recently identified chromosome 9p24.1/programmed cell death-ligands 1/2 (PD-L1/PD-L2) alterations, which result in increased expression of PD-L1 in almost all classical Hodgkin lymphoma cases [19]. Therefore, immunotherapies using PD-L1 blockade have become a new treatment method for classical Hodgkin lymphoma. As for diagnostic methods, it was recently documented that immunohistochemistry for programmed cell death protein 1 (PD-1) and PD-L1 could be helpful to diagnose classical Hodgkin lymphoma with a small amount of biopsy samples [20, 21]. In this case, an external incisional biopsy was necessary to guide early diagnosis and treatment. However, the patient’s atypical clinical presentation and unstable general condition made it difficult for us to perform surgical intervention. In such cases, it is important to obtain detailed immunohistochemical information from a small amount of biopsy material. We hope that PD-1 and PD-L1 immunohistochemistry as a diagnostic method will play an important role in the diagnosis of classical Hodgkin lymphoma in the future.

Hodgkin lymphoma is a relatively rare disease among B-cell lymphomas; however, in recent years, new findings have been accumulated, such as the usefulness of PD-1 and PD-L1 immunohistochemistry for diagnosis, and effectiveness of immunotherapies using PD-L1 blockade. Furthermore, we highlight the fact that even patients with Hodgkin lymphoma, which is generally known as a slow-progressing disease, show a rapid course similar to that of TAFRO syndrome. This case report provides valuable insights toward a deeper understanding of the disease concepts, pathophysiology, and diagnostic methods in Hodgkin lymphoma.

## Conclusion

It is sometimes difficult to diagnose malignant lymphomas using a small amount of biopsy material. Herein, we emphasize the importance of performing an external incisional mass biopsy of the lymph nodes. This incision can guide early diagnosis and treatment of malignant lymphoma when strongly suspected.

## Data Availability

Not applicable.

## Abbreviations

TAFRO syndrome: Thrombocytopenia, anasarca, fever, reticulin fibrosis or renal insufficiency, and organomegaly syndrome
LAHS: Lymphoma-associated hemophagocytic syndrome
iMCD: Idiopathic multicentric Castleman disease
CT: Computed tomography
MRCP: Magnetic resonance cholangiopancreatography
ERCP: Endoscopic retrograde cholangiopancreatography
PD-1: Programmed cell death protein 1
PD-L1: Programmed cell death ligand 1
